# Exosomes as a tumor immune escape mechanism: possible therapeutic implications

**DOI:** 10.1186/1479-5876-6-37

**Published:** 2008-07-22

**Authors:** Thomas E Ichim, Zhaohui Zhong, Shalesh Kaushal, Xiufen Zheng, Xiubao Ren, Xishan Hao, James A Joyce, Harold H Hanley, Neil H Riordan, James Koropatnick, Vladimir Bogin, Boris R Minev, Wei-Ping Min, Richard H Tullis

**Affiliations:** 1 Medistem Laboratories Inc, San Diego, USA; 2 Department of Surgery, Pathology, Oncology, Microbiology and Immunology, University of Western Ontario, London, Canada; 3 Department of Surgery, the Second Xiangya Hospital of Central South University, Changsha, PR China; 4 Department of Ophthalmology, University of Florida, Gainesville, USA; 5 Department of Surgery, Tianjin Medical University, Tianjin, PR China; 6 Aethlon Medical, San Diego, California, USA; 7 Moores UCSD Cancer Centre, San Diego, USA; 8 Division of Neurosurgery, University of California San Diego, San Diego, USA; 9 Medistem Laboratories, 9255 Towne Centre Drive, Suite 450, San Diego, California, 92122, USA

## Abstract

Advances in cancer therapy have been substantial in terms of molecular understanding of disease mechanisms, however these advances have not translated into increased survival in the majority of cancer types. One unsolved problem in current cancer therapeutics is the substantial immune suppression seen in patients. Conventionally, investigations in this area have focused on antigen-nonspecific immune suppressive molecules such as cytokines and T cell apoptosis inducing molecules such as Fas ligand. More recently, studies have demonstrated nanovesicle particles termed exosomes are involved not only in stimulation but also inhibition of immunity in physiological conditions. Interestingly, exosomes secreted by cancer cells have been demonstrated to express tumor antigens, as well as immune suppressive molecules such as PD-1L and FasL. Concentrations of exosomes from plasma of cancer patients have been associated with spontaneous T cell apoptosis, which is associated in some situations with shortened survival. In this paper we place the "exosome-immune suppression" concept in perspective of other tumor immune evasion mechanisms. We conclude by discussing a novel therapeutic approach to cancer immune suppression by extracorporeal removal of exosomes using hollow fiber filtration technology

## Cancer is Recognized by the Immune System

The concept of whether cancer is recognized by the immune system has been a topic of intense discussions and experimentation for more than a century. Philosophically speaking, the argument revolves around one central aspect: cancer originates from "self" tissue, therefore why should the immune system attack it? The traditional concept of immunology which teaches that the main purpose of the immune system is to distinguish between "self" and "non-self" suggests that since cancer is "self" there should be no immune response against it. Current-day immunological advances, however, have struck down this notion. However, before going into these advances in detail, we will first overview the history of cancer immunotherapy in order to provide a background for our discussion.

In the late 1800s a physician at Sloan Kettering Cancer Center, William Coley, made the empirical observation that certain types of tumors would go into remission subsequent to bacterial infections. One of the first patients he saw in his career was a young girl who died of a rapidly progressing sarcoma originating in her arm. A different patient with a similar type of sarcoma in the neck had lived for seven years after diagnosis with no detectable signs of cancer. The only noteworthy difference between the two patients, in his mind was that the latter patient had repeated encounters with bacterial infections. This prompted Coley to search the medical literature, where he found that sarcoma remission had previously been documented to be associated with erysipelas, a streptococcal infection of the skin. This prompted Coley to begin purposely inoculating patients with various bacterial extracts with the aim of stimulating an immune response that would somehow "cross-over" and lead to cancer regression. He reported that the first patient purposely inoculated suffered from an inoperable late stage neck sarcoma. This patient was administered extracts of a streptococcal broth that was generated from another patient. According to the published description, this treatment led to a significant remission of a "hen egg"-sized tumour within ten days, and resulted in patient survival for eight years, after which he died of tumour relapse [1,2]. Eventually, due to the uncontrollable effects of unstandardized bacterial mixtures, Coley generated a combination of heat-killed serratia marcescens and heat-killed streptococci which were eventually named "Coley's Toxins" and sold in the United States by Parke-Davis from 1923 to 1963 [3]. The advent of chemotherapy, as well as unpredictable reactions that patients would have to Coley's Toxin contributed, at least in part, to the discontinuation of this therapy. Nevertheless, William Coley is considered by many the father of modern day cancer immunotherapy [4]. Interestingly, a standardized version of Coley Vaccine is currently being developed by the Canadian biotechnology company MBVax.

Despite the suggestion that immune response to bacterial infections may somehow "re-awaken" the immune system to kill cancer, scientifically, there could have been other explanations for the effect of Coley's Toxins. For example, it may be possible that compounds inside the bacterial extracts had ability to directly kill cancer cells [5], or to preferentially inhibit tumor angiogenesis [6]. Accordingly, in our discussion of whether the immune response actually inhibits cancer or not, we will turn to animal models.

The era of molecular biology has allowed for gene-specific deletion in animals. This means that genes associated with immune responses can be "knocked-out" of animals so as to study the importance of the specific gene in an in vivo setting. Speaking in very general terms, there are two pathways that the immune system can take when it is activated. The first type is called "Th1", which is involved in destroying cells of the body that are infected from the inside, such as virally infected cells. The second type of immune response is called "Th2", which is responsible for killing targets that reside outside of the cells of the body, such as parasites and certain bacteria [7,8]. Since cancer consists of cells of the body that have distinguishing properties from the other cells (ie high proliferation, ability to metastasize, etc), it may be possible to rationalize the Th1 path of the immune response would be the one responsible for control of cancer, if the immune response is involved at all. Indeed, Coley's toxin (and its constituents) were identified decades later to be potent inducers of the Th1 cytokine TNF-alpha, as well as activators of this general immune response pathway [9,10]. The discovery of transcription factors that induce Th1 or Th2 immunity has allowed experimental assessment of the roles of these types of immune responses in control of cancer. Transcription factors controlling Th1 immunity include T-bet [11], and STAT4 [12], and those controlling Th2 include GATA-3 [13] and STAT6 [14,15]. When various tumors are administered to STAT6 knockout animals (therefore having a Th1 predisposition since the Th2 pathway is ablated), these tumors are either spontaneously rejected [16], or immunity to them is achieved with much higher potency compared to wild-type animals [17]. Furthermore, in STAT6 knockout animals, immunologic resistance to metastasis formation is observed [18]. On the other hand, STAT4 knockout mice lack Th1 capability and therefore have only upregulated Th2 immunity. Such animals allow accelerated cancer formation after treatment with chemical carcinogens [19].

While the above suggest the importance of the Th1 immune response in controlling tumors, in many cases animal data does not translate efficiently to human disease. Accordingly, we turn our attention to situations where immune suppression is induced either by genetic abnormality or in response to a medical condition. In general, natural killer (NK) cell activity is associated with Th1 immune responses and tumor immunity [20]. Patients with the congenital abnormality Chediak-Higashi Syndrome, are characterized by absent or severely diminished NK function. In this population, the overall incidence of malignant tumors is 200–300 times greater than that in the general population [21]. Another example of an inborn trait associated with immune deviation is patients born with a specific polymorphism of the IL-4 receptor gene that is known to be associated with augmented Th2 responses. Multivariate regression analysis showed that this polymorphism was an independent prognostic factor for shorter cancer survival and more advanced histopathological grade [22]. In addition to inborn genetic abnormalities, the immune suppressive regimens used to prevent transplant rejection are associated with a selective inhibition of Th1 responses [23-25]. In support of the concept that suppression of Th1 immunity is associated with cancer onset, the incidence of cancer in the post-transplant population is markedly increased in comparison to controls living under similar environmental conditions [26-31]. In terms of disease associated immune suppression, HIV infected patients also have a marked predisposition to a variety of tumors, especially, but not limited to lymphomas, as a result of immunodeficiency [32].

The above examples support a relation between immune suppression (or Th2 deviation) and cancer proliferation, the opposite circumstance, of immune stimulation resulting in anticancer response is also documented. Numerous clinical trials using antigen specific approaches such as vaccination with either tumor antigens alone [33,34], tumor antigens bound to immunogens [35,36], tumor antigens delivered alone [37] or in combination with costimulatory molecules by viral methods [38], tumor antigens loaded on dendritic cells ex vivo [39-41], or administration of in vitro generated tumor-reactive T cells [42], have all demonstrated some, albeit modest clinical effects. It is documented that inappropriate immune responses (broadly speaking Th2 responses) can actually stimulate tumor growth [43,44]. Accordingly, these data all support the presumed recognition of cancer by the immune system and the notion that the immune system, if stimulated properly, may induce cancer regression.

The philosophical question posed at the beginning of this discussion; how can the immune system recognize cancer when cancer is part of self, is answered in the following manner. The immune system is not responsible for seeing only "self" versus "non-self" but actually seeing and responding to different variations of "self". The tumor, in its quest for proliferative advantage, ability to metastasize, and need for formation of new blood supply, actually expresses new molecules at levels that are recognized by the immune system. Immunological recognition of molecules needed for the tumor to have the "cancer phenotype" has been well-documented. We will not provide an overview of these data here but will provide some examples. Specifically, the proliferative advantage of tumors is associated with growth factor receptor upregulation, accordingly immune responses to various such receptors are known to exist naturally or to be inducible [45,46]. The same is true for matrix metalloproteases involved in tumor extravasation and metastasis [47,48], as well as for angiogenic factors involved in formation of new blood vessels [49,50]. The question, of course remains, if the immune system can see cancer, why does it not eradicate it, and why has the clinical implementation of cancer immunotherapy yielded such poor results at the bedside?

## Cancer Suppresses the Immune System

The development of successful immune responses to cancer is hindered by numerous factors, including primarily, that ability of the tumor to cause suppression of a productive host immune response to cancer. The interaction between the tumor and the immune system has been likened to pregnancy, in which an allogeneic graft (the fetus) rapidly develops without rejection by an immunogically competent host. The ability of the fetus to evade the maternal immune response of the mother is not due to anatomical barriers, since maternal immune cells have been demonstrated to cross the placenta and actually enter the fetus [51]. What seems to occur in pregnancy is similar to the cancer patient in that there occurs a selective depletion of immune components, while other immunological parameters are left intact. Both in pregnancy and cancer a specific depletion of certain T cells occurs via numerous common mechanisms such as FasL [52-54]. Before elaborating on specific mechanisms by which FasL kills immune system cells, we will first discuss some of the historical work that led us to the notion that cancer suppresses the immune system.

Experiments in the 1970s demonstrated the existence of immunological "blocking factors", then-unidentified components of plasma found in cancer patients and pregnant women that inhibited antigen-specific lymphocyte responses. Some of this early work involved culturing autologous lymphocytes with autologous tumor cells in the presence of third party healthy serum. This culture resulted in an inhibition of growth of the autologous tumor as a result of the lymphocytes. Third party lymphocytes did not inhibit the growth of the tumor. Interestingly when autologous serum (i.e. from the cancer patient) was added to the cultures, the lymphocyte-mediated inhibition of tumor growth was not observed. These experiments gave rise to the concept of antigen-specific "blocking factors" found in the body of cancer patients that incapacitate successful tumor immunity [55-57]. This work stimulated the more recent demonstration of tumor-suppression of immune function in experiments showing that T cell function is suppressed in terms of inability to secrete interferon gamma due to a cleavage of the critical T cell receptor transduction component, the TCR-zeta chain [58]. Originally, zeta chain cleavage was identified in T cells prone to undergo apoptosis [59]. Although a wide variety of explanations have been put forth for the cleavage of the zeta chain, one particular cause was postulated to be tumor-secreted microvesicles [60]. Since the immune suppressive effects of cancer are systemic, the ability of microvesicles secreted by tumor cells to specifically induce T cell modulation through circulating through-out the body has attracted considerable attention. While there are several known mechanisms of cancer to suppress the immune system that do not use microvesicles, their sheer number in the cancer patient, their ability to systemically influence numerous immune parameters, as well as the fact that administration of cancer microvesicles stimulates cancer progression, all point to their important role in cancer evasions of the immune response.

## Cancer Secreted Microvesicles: A Mechanism of Escaping from the Immune System

In the 1980's Dr. Douglas Taylor described microvesicles secreted by tumor cells [61]. They were estimated to be between 50–200 nanometers in diameter and associated with a variety of immune inhibitory effects. Specifically, it was demonstrated that such microvesicles could not only induce T cell apoptosis, but also block various aspects of T cell signaling, proliferation, cytokine production, and cytotoxicity [62-64]. Although much interest arose in the biology of microvesicles, few therapeutic applications developed since microvesicles were uncharacterized at a molecular level.

Other research identified another type of microvesicular-like structures, which were termed "exosomes". Originally defined as small 80–200 nanometers in diameter, exosomes were observed initially in maturing reticulocytes [65,66]. Subsequently it was discovered that exosomes are a potent method of dendritic cell communication with other antigen presenting cells. Exosomes secreted by dendritic cells were observed to contain extremely high levels of MHC I, MHC II, costimulatory molecules, and various adhesion molecules [67]. In addition, dendritic cell exosomes contain antigens that said dendritic cell had previously engulfed [68]. The ability of exosomes to act as "mini-antigen presenting cells" has stimulated cancer researchers to pulse dendritic cells with tumor antigens, collect exosomes secreted by the tumor antigen-pulsed dendritic cell, and use these exosomes for immunotherapy. Such exosomes were seen to be capable of eradicating established tumors when administered in various murine models [69,70]. The ability of dendritic exosomes to potently prime the immune system brought about the question if exosomes may also possess a tolerance inducing or immune suppressive role. Since it is established that the exosome has a high concentration of tumor antigens, the question arose whether exosomes may induce an abortive T cell activation process leading to anergy [71]. Specifically, it is known that numerous tumor cells, and exosomes derived therefrom, express the T cell apoptosis-inducing molecule FasL [71-73].

FasL is an integral type II membrane protein belonging to the TNF family whose expression is observed in a variety of tissues and cells such as activated lymphocytes and the anterior chamber in the eye. FasL induces apoptotic cell death in various types of cells target cells via its corresponding receptor, CD95/APO1. FasL not only plays important roles in the homeostasis of activated lymphocytes, but it has also been implicated in establishing immune-privileged status in the testis and eye, as well as a mechanisms by which tumors escape immune mediated killing. Accordingly, given the expression of Fas ligand on a variety of tumors, we and others have sought, and successfully demonstrated that FasL is expressed on exosomes secreted by tumor cells [71]. Due to the ability of exosomes to mediate a variety of immunological signals, it was proposed that at the beginning of the neoplastic process, tumor secreted exosomes selectively induce antigen-specific T cell apoptosis, through activating the T cell receptor, which in turn upregulates expression of Fas on the T cell, subsequently, the FasL molecule on the exosome induces apoptosis. This process may be occurring by a direct interaction between the tumor exosome and the T cell, or it may be occurring indirectly by tumor exosomes binding dendritic cells, then subsequently when T cells bind dendritic cells in lymphatic areas, the exosome actually is bound by the dendritic cell and uses dendritic cell adhesion/costimulatory molecules to form a stable interaction with the T cell and induce apoptosis. In the context of more advanced cancer patients, where exosomes reach higher concentrations systemically, the induction of T cell apoptosis occurs in an antigen-nonspecific, but FasL, MHC I-dependent manner.

The recent recognition that tumor secreted exosomes are identical to the tumor secreted microvesicles described in the 1980s [74], has stimulated a wide variety of research into the immune suppressive ability of said microvesicles. Specifically, immunesuppressive microvesicles were identified not only in cancer patients [60,63], but also in pregnancy [75-77], transplant tolerance [78,79], and oral tolerance induction [80,81]. Accordingly, one ideal method of stimulating the immune response of a cancer patient would be the removal of these immunosuppressive microvesicles from circulation through the use of an extracorporeal filter.

## Microvesicle Removal by Extracorporeal Filtration: The Hemopurifier™

Previous efforts have been made to remove cancer associated immune suppressive factors through extracorporeal means. One most prominent example of this is a report by Lentz's group in which 16 patients with metastatic cancers were treated with ultrapheresis, a procedure that removes certain fractions of blood associated with immune suppression. Treatment was associated with a marked increase in lymphocytic infiltration of tumor and tumor necrosis as seen by repeat biopsy. In some patients, immunological anergy was reversed and Karnofsky status improved. Six of the 16 patients had reduction of the sum of mean cross-sectional diameters of measureable lesions by 50% or more [82]. Although this study demonstrated the proof-of-principle that stimulation of cancer immune responses can be clinically performed by extracorporeal removal of immune suppressants, this approach has several limitations: a) not-selective for specific inhibitors; b) theoretically would result in loss of immune stimulatory cytokines; and c) is not applicable on a wide scale.

The San Diego biotechnology company, Aethlon Medical, has developed a novel hollow-fiber cartridge (Hemopurifier™) that is compatible with standard dialysis machines. Recently completed clinical trials have demonstrated its safety in hepatitis patients. Effective removal of HIV particles, which have a similar size to exosomes, was demonstrated using a variety of settings [83-85].

The Hemopurifier™ utilizes an affinity substrate a proprietary lectin-based resin, which possesses high affinity for heavily glycosylated viral surface proteins, which can selectively deplete circulating virus with resultant accumulation of virus in the cartridge. Tumor cell membranes and shed immunosuppressive microvesicles are also highly glycosylated and thus possess a preferential affinity to lectins in comparison to non-malignant cells [86-88]. Cancer patients have a much higher level of circulating microvesicles. The level of microvesicles in cancer patients is 2,000–5,000 ug/ml protein/ml of blood versus 0–0.5 ug in healthy volunteers [62,76]. Given the high concentration of circulating microvesicles, as well as the potential for selected depletion due to glycosylation differences, the Hemopurifier™ in its current state is an attractive candidate for removal of cancer-secreted immunosuppressive microvesicles.

The ability to attach different molecules and antibodies to the resin of the Hemopurifier™ cartridge, increases the potential selective exosome removal targets. For example, clinically used antibodies such as Herceptin, could be attached to the Hemopurifier™ resin in order to deplete microvesicles expressing HER2 protein. Additionally, numerous proteins exist that are found on cancer-secreted microvesicles such as FasL, MHC I, MHC II, CD44, placental alkaline phosphatase, TSG-101, MHC I-peptide complexes, MHC II-peptide complexes. Antibodies to these proteins are commonly available and can be incorporated into the Hemopurifier™ cartridge with little effort.

## Conclusion

The Hemopurifier™ cartridge can enter the cancer therapeutics arena with relative ease due to several factors. Firstly, the cartridge is already produced under Good Manufacturing Practices with clinical safety data available, albeit for different indications. Secondly, the cartridge is compatible with standard dialysis systems which are present at every major medical institution. Thirdly, the patient population whose treatment is proposed with this novel approach has few or no treatment alternatives. Fourthly, the Hemopurifier™ cartridge can be used not only as a monotherapy, but also as an adjuvant to currently used immunotherapeutic approaches. The commercialization of therapies as adjuvants to existing therapies is well-accepted in the industry, as seen in the approval of Herceptin and Avastin for use with specific chemotherapeutic agents.

In conclusion, the use of the Hemopurifier™ as a means of de-repressing immune functions in cancer patients is a novel, easily implemented approach to cancer therapeutics that will not only provide an alternative to the currently ineffective approaches, but also provide a framework for development of strategies that may benefit cancer patients in a multidimensional manner.

## Authors' contributions

TEI, ZZ, SK, XZ, XR, XH, JAJ, HHH, NHR, JK, VB, MRM, WP, and RHT were involved in conceiving the paper and reducing it to practice.
